# Biliary Ascariasis on Magnetic Resonance Cholangiopancreatography

**DOI:** 10.4103/0974-777X.56248

**Published:** 2009

**Authors:** Mohammad A Hashmi, Jevan K De

**Affiliations:** *EKO CT and MRI Scan Centre, At Medical College and Hospitals Campus, 88-College Street, Kolkata-700 073, India*; 1 *Medical College and Hospitals, Kolkata*

**Keywords:** Ascaris lumbricoides, Common bile duct, Magnetic resonance cholangi-pancreatography

## Abstract

A 17-year-old girl presented with features of biliary obstruction. Magnetic resonance cholangi-pancreatography revealed typical linear signals in common bile duct, which appears like Ascaris lumbricoides. The diagnosis was confirmed by endoscopic removal of the worm.

## INTRODUCTION

Parasite infecion of the biliary tract is a common complication. *Ascaris lumbricoides, Clonorchis sinensis, Opisthorchis viverrini, Opisthorchis felineus,* and *Dicrocoelium dendriticum* are closely related to *C. sinensis* and can also cause serious biliary complications. Fascioliasis, caused by *Fasciola hepatica* and *F. gigantica*, is a zoonotic helminthiasis that can present as acute hepatic or chronic biliary tract infection.[1] Ascaris lumbricoides is the most common cause of parasitic infection of bile duct.[2–4] Occasionally, the adult *Ascaris* worm may cross into vater's ampulla and enter the bile duct, gall bladder or pancreatic duct, leading to a variety of complications such as biliary colic, gallstone formation, cholecystitis, pyogenic cholangitis, liver abscess and pancreatitis. Computed tomography (CT), Magnetic resonance imaging (MRI), and ultrasound are useful imaging tools to identify these parasites and their complications. Recently, MRCP has been playing a significant role in diagnosing biliary infection by Ascaris.

## CASE REPORT

**Figure 1:**
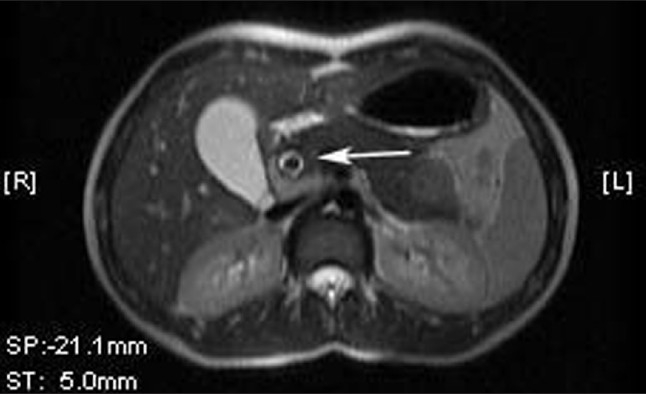
T2 weighted axial image is showing hypointense signal in CBD. Adjacent bile appears hyperintense in CBD

**Figure 2:**
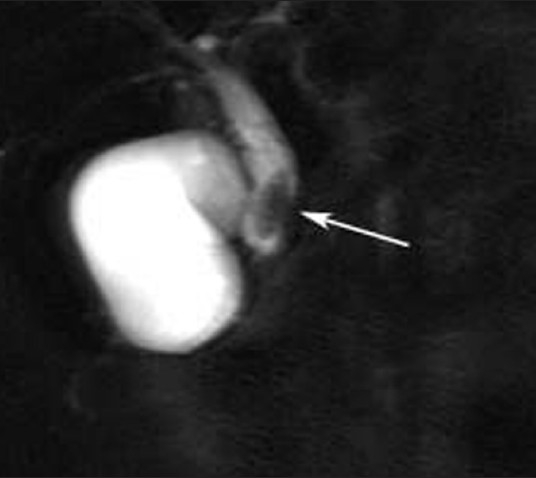
MRCP single shot shows linear signal of ascariasis in CBD

**Figure 3:**
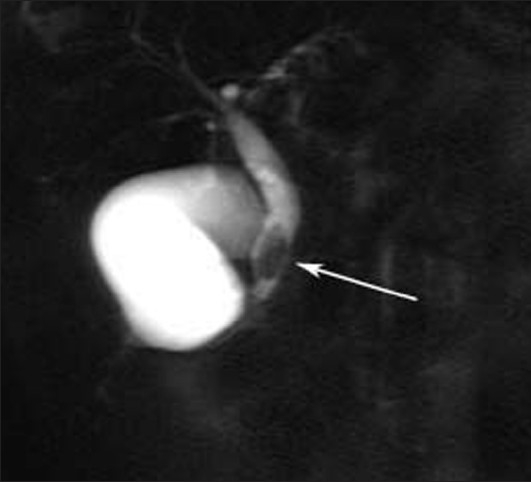
MRCP single shot shows above findings as in Figure 2 but with slight different angle

A 17-year-old female presented to us with pain abdomen. Patient liver function test showed mild changes. Ultrasound had shown few linear echogenicites in common bile duct (CBD). MRI revealed hypointense dot type signal in center of CBD in axial imaging [Figure 1]. Single shot MRCP showed linear hypointense signal in CBD [Figures 2 and 3]. The worm was removed endoscopically and the patient improved gradually.

## DISCUSSION

Ascaris lumbricoides is one of the most common parasitic infections worldwide. An adult worm is typically 15-50 cm long and 3-6 mm thick. During the intestinal phase the worms may be silent or cause abdominal pain, vomiting or bowel obstruction. Migration of a worm through the papilla of vater into the biliary tree is an uncommon complication and leads to biliary colic, recurrent pyogenic cholangitis, pancreatitis, hepatic abscesses and septicemia.[4,5,7] These irritating factors can even lead to cholangiocarcinoma.[1]

Ulttrasound (US) is the imaging modality of choice for biliary problems. US findings of biliary ascariasis have been described as tubular, echogenic, non-shadowing structures, sometimes with a thin, longitudinal, central sonolucent line. Movement of worms can also be seen.[2,7]

MRI and MRCP are good imaging modalities to detect the above condition. Axial images in T2 weighted sequence shows a dot hypointense signal in CBD around which the bile signals are hyperintense. MR cholangiography shows intraductal worms as linear hypointense filling defects.

## CONCLUSION

US, CT and other modalities can detect biliary Ascariasis while MRI with MRCP is very effective in detecting intraductal Ascariasis. They can be seen as linear hypointensities intraluminally along the course of CBD.
